# Supplementary material for: A branched heterochronic pathway directs juvenile-to-adult transition through two LIN-29 isoforms
Source: eLife. 2020 Mar 30;9:e53387. doi: 10.7554/eLife.53387 (PMC7105380; doi:10.7554/eLife.53387)
Supplement: Supplementary file 1. [file elife-53387-supp1.pdf]

Supplementary file 1A: Plasmid used in this study

Short name	Plasmid backbone	Insert	Cloning technique	Primers[a] or gBlocks[b]	
pIK155	published in (Katic et al., 2015)				
pIK198	published in (Katic et al., 2015)				
pCFJ90	published in (Frøkjær-Jensen et al., 2008)				
pCFJ104	published in (Frøkjær-Jensen et al., 2008)				
pFA27	published in (Aeschimann et al., 2019)				
pFA224	pENTR L4-R1	*col-10* promoter	gift from Matyas Ecsedi. The col-10 promoter starts is 1320 bp long and starts with the following sequence: 5'-CCTCGGGTGGTCATCATCTCAAATTCCAATCTTCTTTTTCATTTTCAATCTCTAACAATTTCAAATT ACTAGAACCCACAAACCAATGTTCGATA … – 3'		
pFA198	pENTR L1-L2	3xFLAG tag followed by BamHI restriction site (can be used for Gibson assembly instead of BP reaction)	BP reaction[c]	fwd primer	ggggacaagtttgtacaaaaaagcaggctATGGATTATAAAGACGATGACGATAAGCGTGA
				rev primer	ggggaccactttgtacaagaaagctgggtGGATCCTCTCTTGTCATCGTCATCCTTGTAATC
pFA199	pENTR L1-L2	3xFLAG::*lin-29a* (ORF and 3'UTR; including introns except for intron 4)	Gibson assembly[d]	fwd(1)	acaaggatgacgatgacaagagaATGGATCAAACTGTTCTAGATTCGGCA
				rev(1)	gcttttgttcgaaCTGTGGATATCTTCCAAATTGTGA
				fwd(2)	tggaagatatccacagTTCGAACAAAAGCCGGACGT
				rev(2)	tgcccactttgtacaagaaagctgggtCCACTGTGCATGAAAAAGCTGGT
pFA206	pENTR L1-L2	3xFLAG::*lin-29b* (ORF and 3'UTR, including introns)	Gibson assembly[e]	fwd primer	acaaggatgacgatgacaagagaATGCAGATGCGGGAAGCAAAAC
				rev primer	tgcccactttgtacaagaaagctgggtCCACTGTGCATGAAAAAGCTGGT
pFA207	pENTR L1-L2	3xFLAG::*mab-10* (ORF and 3'UTR, including introns)	Gibson assembly[e]	fwd primer	acaaggatgacgatgacaagagaATGTCATCATCGTCGTCGTC
				rev primer	tgcccactttgtacaagaaagctgggtACTATTGTTACGGGAATCATGTCT
pFA218	published in (Aeschimann et al., 2019)				
pFA219	pENTR L1-L2	FLAG-HA-degron::*lin-29a* (ORF and 3'UTR; including introns except for intron 4)	Gibson assembly[f]	fwd primer	aggcggcggcgttcgtgaagATGGATCAAACTGTTCTAGATTCGGCA
				rev primer	tgcccactttgtacaagaaagctgggtCCACTGTGCATGAAAAAGCTGGT
pFA220	pENTR L1-L2	FLAG-HA-degron::*lin-29b* (ORF and 3'UTR, including introns)	Gibson assembly[f]	fwd primer	aggcggcggcgttcgtgaagATGCAGATGCGGGAAGCAAAAC
				rev primer	tgcccactttgtacaagaaagctgggtCCACTGTGCATGAAAAAGCTGGT
pFA221	pENTR L1-L2	FLAG-HA-degron::*mab-10* (ORF and 3'UTR, including introns)	Gibson assembly[f]	fwd primer	aggcggcggcgttcgtgaagATGTCATCATCGTCGTCGTC
				rev primer	tgcccactttgtacaagaaagctgggtACTATTGTTACGGGAATCATGTCT
pENTR_R2-L3_operon-GFP-H2b	published in (Ecsedi et al., 2015) based on (Merritt et al., 2008)				
pFA238	pCFJ150	pFA224, pFA219, pENTR_R2-L3_operon-GFP-H2b	LR reaction		
pFA239	pCFJ150	pFA224, pFA220, pENTR_R2-L3_operon-GFP-H2b	LR reaction		
pFA240	pCFJ150	pFA224, pFA221, pENTR_R2-L3_operon-GFP-H2b	LR reaction		

[a]Overhangs are in lowercase, the part annealing to the template in uppercase. If not stated otherwise, the PCR products were amplified from *C. elegans* genomic DNA.

[b]Overhangs for Gibson assembly reactions are in lowercase.

[c]PCR product was amplified from pFA27.

[d]Assembly with BamHI-digested plasmid pFA198 and two PCR products.

[e]Assembly with BamHI-digested plasmid pFA198 and one PCR product.

[f]Assembly with BamHI-digested plasmid pFA218 and one PCR product.

Supplementary file 1B: oligonucleotides and sgRNAs used in this study

Identifier	Sequence	Source	Description
CA1-2	ctaagcctaagtctatgcctaagcccaagcttgatctcaca atttaagctttcaaactagtgctaattaaag ggaatacggtttcagaattaaggagacacctagatcaatt gagctctaaagatccactatattcaatgtac ttacagttcgaacaaaagccggacgtgggggtgcttcag caacagatgcagatgcgg	This study	HR oligo for generation *lin-29(xe61 xe114)* and *lin-29(xe116)*
FA419	gaacggaatgatccgaaacctagatcaattgagctctaa agatccactatattcaatgtacttacagttc gaacaaaagccagatgtcggggtgcttcagcaacagat gcagatgcgggaagcaaaaccttacaa gtgcacgcagtgtgtcaaggtatgtggt	This study	HR oligo for generation *lin-29(xe61 xe121)* and *lin-29(xe120)*
FA434	cctttcagcttcgcgaggatcaagccaacttcttcaacgca atgttttcacaatttggaagatatccacag gtttgcatagggaacacattcaaacgagggtga	This study	HR oligo for generation *lin-29(xe61 xe133)* and *lin-29(xe200)*
3-AC15-16	acagaattaaggagacaagg	This study	sgRNA for generation *lin-29(xe61 xe114)* and *lin-29(xe116)*
2-AC2-3	agctcaattgatctaggttt	This study	sgRNA for generation *lin-29(xe61 xe114)* and *lin-29(xe116)*
L29_ATG1	gttcgaacaaaagccggacg	This study	sgRNA for generation *lin-29(xe61 xe121)* and *lin-29(xe120)*
L29_ATG3	agctgaagcacccccacgtc	This study	sgRNA for generation *lin-29(xe61 xe121)* and *lin-29(xe120)*
L29_031	gctggaaccaccactggctc	This study	sgRNA for generation *lin-29(xe61 xe133)* and *lin-29(xe200)*
L29_041	gtggcaggagagaattctga	This study	sgRNA for generation *lin-29(xe61 xe133)* and *lin-29(xe200)*
L29_if1	agccaacttcttcaacgcaa	This study	sgRNA for generation *lin-29(xe61 xe133)* and *lin-29(xe200)*
L29_if3	gtgaaaacatatgatgtggc	This study	sgRNA for generation *lin-29(xe61 xe133)* and *lin-29(xe200)*
lin-29_F4	ccagcacatcattcgatcact	(Aeschimann et al., 2017)	qPCR primer for *lin-29a* specific detection (exon3-exon4 junction)
lin-29_R4	gaagttcagtagatccgcttga	(Aeschimann et al., 2017)	qPCR primer for *lin-29a* specific detection (exon3-exon4 junction)

lin-29_F5	acccaagtttgagttcgaaca	(Aeschimann et al., 2017)	qPCR primer for *lin-29b* specific detection (SL1-exon5 junction)
lin-29_R5	gatgagttggcaaatgccttga	(Aeschimann et al., 2017)	qPCR primer for *lin-29b* specific detection (SL1-exon5 junction)
JKq17	gtcggaagaccacgtcatcaa	This study	qPCR primer for *act-1* specific detection (exon1)
JKq18	agggtaaggatacctctcttgga	This study	qPCR primer for *act-1* specific detection (exon1-exon3 junction)
